# Effects of GRK5 and ADRB1 polymorphisms influence on systolic heart failure

**DOI:** 10.1186/s12967-015-0402-7

**Published:** 2015-02-01

**Authors:** Sheng Kang, Xuan Hong, Chang-wu Ruan, Ping Yu, Shan-shan Yu, Ming Chen, Dai-fu Zhang, Hui-min Fan, Zhong-min Liu

**Affiliations:** Department of cardiology, Shanghai East hospital, Tongji University, Jimo Rd 150, 200120 Shanghai, P. R. China; Department of Cardiology, Shanghai the 8th People’s Hospital, Caobao Road 8, 200235 Shanghai, China; Department of Clinical Laboratory, Shanghai East Hospital, Tongji University, Jimo Rd 150, 200120 Shanghai, P. R. China; Department of Cardiology, Pudong New District People’s Hospital, Chuan Ring Road 490, 201299 Shanghai, China

## Abstract

**Background:**

G-protein receptor kinase 5 (GRK5) Gln41 > Leu and β1-adrenergic receptor (ADRB1) Arg389 > Gly polymorphisms presented the different distribution of genotype frequencies between Caucasian American and African American, and produced the difference in β-blocker treatment effect among them with systolic heart failure (SHF).

**Objective:**

This study sought to identify the distributed characteristics of these variant genotypes in Chinese population, and influence of GRK5 and ADRB1 polymorphisms on SHF morbidity and β-blocker treatment effect in patients with SHF.

**Methods:**

This study was based on cross-sectional survey data. 1794 and 1718 subjects’ ADRB1 and GRK5 gene sequencing (sanger method) data were achieved respectively. Blood samples collection, clinical laboratory detection, electrocardiogram and echocardiography examinations were performed. Medication usage was confirmed at in-hospital visits or the questionnaire by personal interview.

**Results:**

GRK5 Leu41Leu genotype was not found in our Chinese population. In non-SHF population, allele frequencies of GRK5 Gln41 and Leu41 were 2782 (0.992) and 22 (0.008) (Hardy-Weinberg equilibrium test χ^2^ = 0.088, P = 0.767), and allele frequencies of ADRB1 Arg389 and Gly389 were 2127 (0.715) and 849 (0.285) (χ^2^ = 0.272, P = 0.602). In SHF patients, allele frequencies of Gln41 and Leu41 were 446 (0.991) and 4 (0.009) (χ^2^ = 0.018, P = 0.893), and allele frequencies of Arg389 and Gly389 were 331 (0.726) and 125 (0.274) (χ^2^ = 1.892, P = 0.169). Further in logistic regression model, these ADRB1 and GRK5 variants were not significantly independently associated with the risk of SHF morbidity. Those carrying genotype ADRB1 Gly389Gly did not reduce significantly the risk of SHF morbidity after β-blocker therapy.

**Conclusions:**

GRK5 Leu41Leu genotype was not found in our Chinese population, neither ADRB1 nor GRK5 variants presented independently associated with the risk of SHF morbidity, most ADRB1 and GRK5 polymorphisms did decrease significantly the risk of SHF morbidity after β-blocker therapy except for those carrying genotype ADRB1 Gly389Gly.

## Introduction

Heart failure (HF) is an incurable syndrome arising from multiple causes that results in high morbidity rate, mortality rate and healthcare costs [1-3]. The management of HF is also complicated by disease heterogeneity in both inherited genetic cardiomyopathies [4] and the more common nonfamilial dilated and ischemic cardiomyopathies [5,6]. Thus efforts are underway to identify additional genetic markers that will indicate prognosis and guide management of patient care.

Pharmacogenomic interactions involving genetic variants of catecholamine receptors or their effectors (i.e., G-protein receptor kinase 5 helps to terminate β-adrenergic receptor signaling by phosphorylating and uncoupling agonist-occupied receptors from their Gas signal transducers) [7] would be especially relevant given their important role in clinical disease management. Importantly, β adrenergic antagonism (β-blocker), which is a class I treatment indication for HF [8].

Previous pharmacogenomic studies have proposed that the β1-adrenergic receptor (ADRB1) Arg389 > Gly [9] and G-protein receptor kinase 5 (GRK5) Gln41 > Leu [10] polymorphisms, both of which are over-represented in African Americans, may play roles in determining individual clinical responses to β-blockade treatment with HF. Human studies have been inconsistent as to whether there are meaningful associations between this polymorphism and HF outcome [11-13].

Considering that ADRB1 and GRK5 polymorphisms were represented differently among ethnicities, and influenced the β-blocker treatment effect with systolic heart failure (SHF), thus our study was designed to identify whether GRK5 Gln41Leu and ADRB1 Arg389Gly polymorphisms would affect on SHF in Chinese population.

## Method

### Study subjects

Our study was based on 2095 subjects’ cross-sectional survey data (including seven patients with SHF) which has been reported previously [14,15], and other 253 patients with SHF derived from Shanghai East hospital, Pudong new district people’s hospital, and Shanghai the 8^th^ people’s hospital. After excluding some subjects with the missing partial clinical data and/or the blood samples. Finally, 1800 subjects’ blood samples were delivered for ADRB1 and GRK5 polymorphisms sequencing. 1794 and 1718 subjects’ ADRB1 and GRK5 gene sequencing data were achieved respectively. The study protocol was approved by the Institutional Review Board of the Shanghai East hospital affiliated with Tongji university (No, 2010020), and subjects gave written informed consent. Our study was performed to conform the declaration of Helsinki.

Enrollment criteria for SHF were age of 30–75 years, a left ventricular ejection fraction (LVEF) less than 50% or two-dimensional echocardiography of mild or greater systolic dysfunction on visual assessment in multiple views [16] and class II-IV heart failure (new York Heart Association). Excluding criteria for SHF were the final stage of multiply organ dysfunction, severe anaemia, hyperthyreosis, psychosis, pregnancy and lactation.

Blood samples collection, clinical laboratory detection, electrocardiogram examination, echocardiography examination and relevant quality controls were described previously in our two studies [14,15]. Medication usage was confirmed at in-hospital visits or the questionnaire by personal interview.

### Genotyping

Genomic deoxyribonucleic acid (DNA) for genotyping was isolated and extracted using KAPA Express Extract DNA kit (Kapa Biosystems Co, Boston). Homo sapiens adrenoceptor Beta 1 (ADRB1) single nucleoide polymorphism fragment (468 bp) 1165 C is replaced with G, which causes a nonconservative amino acid substitution of Gly for Arg at residue 389 [17], and Homo sapiens G protein-coupled receptor kinase 5 (GRK5) single nucleoide polymorphism fragment (399 bp) 122 A is replaced with T, which results in a nonconservative amino acid substitution of Leu for Gln at position 41 [10].

The PCR primers were designed using Primer 5.0 software (Premier Co, Canada), and PCR primer sequences were screened across the human genome using the National Center for Biotechnology Information Blast program to ensure their specificity for the gene of interest, (http://www.ncbi.nlm.nih.gov/tools/primer-blast/index.cgi?LINK_LOC=BlastHome website).

**Figure 1:**
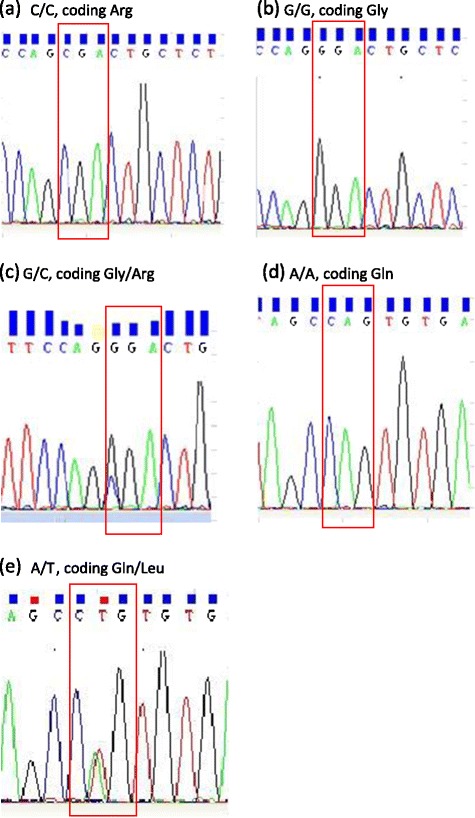
**The major gene sequencing parameters.** Note: The red box indicated ADRB1 codon for coding relevant amino acid at position 389 (Figure **a**-**c**), and GRK5 codon for coding relevant amino acid at position 41 (Figure **d**,**e**).

Forward Primer for ADRB1-F: 5’-CAAGACGCTGGGCATCATCA-3’; Reverse Primer for ADRB1-R: 5’-CCCTACACCTTGGATTCCGA-3’; Forward Primer for GRK5-F: 5’-GTGTGTCTTGGGCTGGGGGT-3’; Reverse Primer for GRK5-R: 5’-AGCCCCTACCTG GAAGCACA -3’, the PCR primers were designed and synthetised by Shanghai branch of BGI Sequencing corporation. The DNA segments containing the region of interest were amplified with the polymerase chain reaction (PCR). GRK5 and ADRB1 PCR procedure (PCR S-5000 thermal cycler, BIO-RAD Co, California, USA) and gene sequence procedure (ABI 3730x1 Applied Biosystem, Applied Biosystem Inc, USA) were also completed by Shanghai branch of BGI Sequencing corporation. The results of gene sequence were presented in Figure 1.

### Statistical analysis

Data analysis was performed with SPSS (version 16.0 for Windows; SPSS Inc, Chicago) and stata 10.0 software (StataCorp, Texas). The normal distributions of numerical variables were described as mean ± SD. Categorical variables were expressed as percentage or proportion. One-way ANOVA and Chi-square test were used to assess significant differences of variables between genotype classes and between SHF group and non-SHF group. If the skew distributed data were expressed as median and quartile, Kruskal-Wallis H test and Mann–Whitney test were used for comparison of clinical variables between genotype classes and between SHF group and non-SHF group. The Hardy-Weinberg equilibrium was assessed using Chi-square test in each genotype group separately. Binary Logistic Regression was performed to assess the risk of SHF morbidity related to GRK5 and ADRB1 polymorphisms. A 2-tailed value of P < 0.05 was considered statistically significant.

## Result

In the present study, 1794 and 1718 of subjects’ ADRB1 and GRK5 gene sequencing data were obtained respectively. In 1796 of subjects in non-SHF population, the recorded genotype frequencies of GRK 5 Gln41Gln, Gln41Leu and Leu41Leu were 1380 (0.984), 22 (0.016) and 0 (0) respectively, allele frequencies of Gln41 and Leu41 were 2782 (0.992) and 22 (0.008), and genotypes were in Hardy-Weinberg equilibrium (χ^2^ = 0.088, P = 0.767). Similarly, the genotype frequencies of ADRB1 Arg389Arg, Arg389Gly and Gly389Gly were 756 (0.508), 615 (0.413) and 117 (0.079) respectively, allele frequencies of Arg389 and Gly389 were 2127 (0.715) and 849 (0.285), and genotypes were in Hardy-Weinberg equilibrium (χ^2^ = 0.272, P = 0.602).

In 260 of SHF patients, the recorded genotype frequencies of GRK5 Gln41Gln, Gln41Leu and Leu41Leu were 221 (0.982), 4 (0.018) and 0 (0) respectively, allele frequencies of Gln41 and Leu41 were 446 (0.991) and 4 (0.009), and genotypes were in Hardy-Weinberg equilibrium (χ^2^ = 0.018, P = 0.893). In the same way, the genotype frequencies of ADRB1 Arg389Arg, Arg389Gly and Gly389Gly were 116 (0.509), 99 (0.434) and 13 (0.057) respectively, allele frequencies of Arg389 and Gly389 were 331 (0.726) and 125 (0.274), and genotypes were in Hardy-Weinberg equilibrium (χ^2^ = 1.892, P = 0.169).

Noticeably, we did not found significantly different of SHF morbidity between genotype classes (see Table 1). Further analysis indicated that age, heart rate, smoking, hypertension, diabetes mellitus, coronary heart disease, chronic renal disease, valvular heart disease, glycosylated hemoglobin and Hs-CRP were closely associated with SHF morbidity, however, ADRB1 and GRK5 variants did not present significantly independently associated with the risk of SHF morbidity (see Table 2). Furthermore GRK5 and ADRB1 polymorphisms did not present significantly difference between non-SHF group and SHF group (see Table 3). Importantly, the two functional β-adrenergic receptor signaling polymorphisms in our population showed not significantly different of genotype frequency distribution and allele frequency distribution between non-SHF group and SHF group, thus this study suggested that GRK5 and ADRB1 polymorphisms did not induce the risk of SHF morbidity.

**Table 1:** **Clinical characteristics of the study population between the genotype groups**

**Characteristic**	**GRK5 Gln41Gln (n = 1681)**	**GRK5 Gln41Leu (n = 26)**	**P value**	**ADRB1 Arg389Arg (n = 907)**	**ADRB1 Arg389Gly (n = 749)**	**ADRB1 Arg389Gly (n = 129)**	**P value**
Age, yrs	58.70 ± 11.34	59.12 ± 9.62	0.852	58.71 ± 11.20	58.84 ± 11.38	56.61 ± 11.99	0.114
Male gender, %	35.7	50.0	0.131	37.2	34.7	47.3	0.023
Weight, kg	62.78 ± 11.04	66.24 ± 8.93	0.119	63.12 ± 10.84	62.60 ± 11.59	63.50 ± 9.76	0.528
Height, cm	161.24 ± 7.92	161.50 ± 8.56	0.868	161.77 ± 7.75	160.82 ± 7.92	163.35 ± 8.27	0.001
Systolic BP, mmHg	133.36 ± 22.43	137.88 ± 17.93	0.316	133.56 ± 23.67	132.95 ± 20.41	132.04 ± 21.14	0.711
Diastolic BP, mmHg	75.71 ± 11.79	79.04 ± 10.96	0.161	75.89 ± 11.83	75.51 ± 11.64	74.75 ± 11.09	0.543
Heart rate, beats/min	74.45 ± 11.38	79.29 ± 12.51	0.039	74.00 ± 10.53	75.06 ± 11.42	71.28 ± 13.90	0.002
Smoking, %	23.6	30.8	0.393	23.7	22.1	29.2	0.199
Drinking, %	14.2	19.2	0.467	13.7	14.2	20.8	0.099
Hypertension, %	39.4	53.8	0.136	38.8	39.9	35.4	0.614
CAD, %	9.3	7.7	0.776	9.5	9.0	5.0	0.470
Diabetes mellitus, %	11.5	15.4	0.542	11.9	11.0	8.5	0.496
SHF, %	13.8	15.4	0.817	13.3	13.9	10.0	0.490
Medication, n (%)							
ACE inhibitors	62 (3.7)	2 (7.7)	0.254	35 (3.8)	22 (2.9)	8 (6.2)	0.174
Angiotensin receptor blockers	156 (9.3)	3 (11.5)	0.729	69 (7.6)	69 (9.2)	12 (8.0)	0.456
Beta-blockers	122 (7.3)	5 (19.2)	0.039	58 (6.4)	61 (8.1)	5 (3.8)	0.132
Aldosterone antagonists	142 (8.4)	2 (7.7)	1.000	69 (7.6)	67 (9.0)	9 (6.9)	0.520
Diuretics	176 (10.5)	2 (7.7)	1.000	89 (9.8)	79 (10.6)	12 (9.2)	0.825
Digoxin	60 (3.6)	0	1.000	36 (4.0)	20 (2.7)	4 (3.1)	0.347
Echocardiography							
LAD, mm	35.58 ± 6.78	34.58 ± 4.21	0.474	35.63 ± 7.09	35.37 ± 6.32	35.84 ± 5.89	0.660
LVESD, mm	30.68 ± 7.41	29.54 ± 3.32	0.451	30.75 ± 7.78	30.25 ± 6.76	30.77 ± 6.60	0.383
LVEDD, mm	47.61 ± 6.40	47.29 ± 4.56	0.805	47.86 ± 6.62	47.39 ± 6.02	48.47 ± 5.66	0.126
LVEF, %	61.51 ± 9.46	62.68 ± 7.40	0.540	61.80 ± 9.37	61.64 ± 9.22	61.62 ± 10.22	0.941
Laboratory							
Total cholesterol, mmol/l	5.02 ± 1.15	4.73 ± 1.21	0.198	5.00 ± 1.09	5.04 ± 1.23	4.84 ± 1.05	0.172
LDL-C, mmol/l	3.03 ± 1.10	2.90 ± 0.88	0.539	3.04 ± 1.06	3.02 ± 0.90	2.85 ± 0.74	0.190
HDL-C, mmol/l	1.29 ± 0.34	1.19 ± 0.31	0.127	1.27 ± 0.35	1.31 ± 0.34	1.26 ± 0.32	0.351
Triglyceride, mmol/l	1.41 [1.00, 2.01]	1.64 [1.67, 2.19]	0.838	1.40[0.99, 2.00]	1.39 [1.01, 2.00]	1.41 [1.00, 2.01]	0.988
Glycosylated hemoglobin, %	5.80 [5.50, 6.20]	5.70 [5.38, 6.25]	0.623	5.70 [5.50, 6.20]	5.80 [5.50, 6.20]	5.64 [5.40, 6.00]	0.041
Creatinine, umol/l	73.46 ± 25.31	78.20 ± 15.35	0.351	73.20 ± 22.38	74.12 ± 28.14	72.54 ± 21.80	0.678
eGFR, ml/min/1.73 m^2^	97.56	86.26	0.078	98.38	96.47	99.47	0.209
Median (Q1,Q3)	[83.59, 113.45]	[76.26, 112.03]		[83.13, 113.22]	[82.18, 113.32]	[86.83, 119.79]	
Plasma NT-proBNP, pg/ml	52.00	45.00	0.867	53.00	51.00	55.00	0.926
Median (Q1,Q3)	[30.00, 87.00]	[33.00, 102.00]		[30.00, 87.00]	[29.00, 91.00]	[28.00, 103.00]	

Note: *ACE* inhibitor, angiotensin converting enzyme inhibitor; *BP*, blood pressure; *CAD*, coronary artery disease; *eGFR*, glomerular filtration rate (ml/min/1.73 m^2^) = 175 × creatinine^-1.234^ × age^-0.179^ × 0.79 (if female); HDL-C, high-density lipoprotein cholesterol; *LAD*, Left atrial diameter; *LDL*-*C*, low-density lipoprotein cholesterol; *LVEF*, left ventricular ejection fraction; *LVESD*, left ventricular end-systolic dimension; *LVEDD*, left ventricular end-diastolic dimension; *NT*-*ProBNP*, *N*-terminal pro-B-type natriuretic peptide; *SHF*, systolic heart failure.

**Table 2:** **Clinical analysis in multivariable clinical model**

**Variable**	**OR**	**95% CI**	**P value**
Age	1.123	1.074 to 1.174	0.000
Male gender	1.166	0.321 to 4.231	0.851
BMI	0.983	0.942 to 1.026	0.435
Heart rate	1.029	1.003 to 1.055	0.025
Smoking	2.734	1.026 to 7.291	0.044
Drinking	1.287	0.908 to 1.824	0.157
Hypertension	3.277	2.500 to 4.296	0.000
Diabetes mellitus	1.844	1.102 to 3.085	0.020
Coronary heart disease	9.702	6.056 to 15.543	0.000
Valvular heart disease	8.107	4.594 to 14.307	0.000
Chronic renal disease	2.045	1.140 to 3.668	0.016
Glycosylated hemoglobin	1.083	1.009 to 1.163	0.027
Hs-CRP	1.087	1.048 to 1.128	0.000
GRK5	2.377	0.237 to 23.870	0.872
GRK5			
Gln41Gln	Reference	Reference	
Gln41Leu	2.219	0.216 to 22.812	0.503
ADBR1	1.121	0.608 to 2.064	0.715
ADRB1			
Arg389Arg	Reference	Reference	
Arg389Gly	1.478	0.638 to 3.424	0.362
Gly389Gly	0.820	0.157 to 4.284	0.814

Note: Significant factors (p < 0.05) for the risk of SHF. BMI, body mass index (kilogram/meter^2^) = weight/height^2^; CI, confidence interval; Hs-CRP (mg/L), high-sensitivity C-reactive protein; OR, odd ratio; other abbreviations as in Table 1. GRK5 and ADRB1 polymorphisms were respectively analyzed as numerical variables and categorical variables for the risk of SHF morbidity.

**Table 3:** **Clinical and genotypic characteristics between non**-**SHF group and SHF group**

**Characteristic**	**Non-SHF group (n = 1976)**	**SHF group (n = 260)**	**P value**
Age, yrs	57.05 ± 9.77	71.06 ± 11.88	0.000
Male, %	634 (32.3)	154 (59.2)	0.000
Weight, kg	62.48 ± 10.39	65.22 ± 14.03	0.006
Height, cm	160.81 ± 7.67	164.66 ± 8.98	0.000
Smoking, %	413 (21.1)	87 (33.5)	0.000
Drinking, %	268 (13.7)	44 (16.9)	0.156
Systolic BP, mmHg	133.87 ± 22.07	131.57 ± 23.17	0.124
Diastolic BP, mmHg	75.76 ± 11.77	76.27 ± 12.08	0.522
Heart rate, beats/min	73.56 ± 11.16	79.17 ± 9.83	0.000
Echocardiography			
LAD, mm	34.19 ± 4.86	46.72 ± 9.40	0.000
LVESD, mm	28.95 ± 4.18	43.60 ± 13.20	0.000
LVEDD, mm	46.66 ± 4.28	56.71 ± 12.41	0.000
LVEF, %	65.00 ± 3.08	39.15 ± 7.79	0.000
GRK5			0.817
Gln41Gln, n (%)	1380 (98.4)	221 (98.2)	
Gln41Leu, n (%)	22 (1.6)	4 (1.8)	
ADRB1			0.490
Arg389Arg, n (%)	756 (50.8)	116 (50.9)	
Arg389Gly, n (%)	615 (41.3)	99 (43.4)	
Gly389Gly, n (%)	117 (7.9)	13 (5.7)	
Medication, n (%)			
ACE inhibitors	47 (2.4)	30 (11.5)	0.000
Angiotensin receptor blockers	117 (6.0)	66 (25.4)	0.000
Beta-blockers	66 (3.4)	87 (33.6)	0.000
Aldosterone antagonists	3 (0.2)	164 (63.1)	0.000
Diuretics	17 (0.9)	188 (72.3)	0.000
Digoxin	3 (0.2)	69 (26.5)	0.000
Statins	36 (1.8)	127 (48.8)	0.000
Nitrates	13 (0.7)	145 (55.8)	0.000

Note: *BP*, blood pressure; *LAD*, Left atrial diameter; *LVEF*, left ventricular ejection fraction; *LVESD*, left ventricular end-systolic dimension; *LVEDD*, left ventricular end-diastolic dimension; SHF, systolic heart failure.

**Figure 2:**
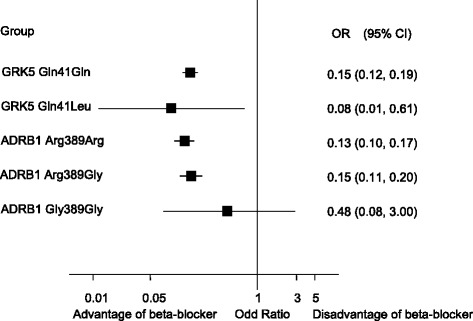
**GRK5 and ADRB1 polymorphisms influence on beta-blocker treatment with SHF.** Note: CI denotes confidence interval. Advantage of beta-blocker indicated that the gene polymorphisms could decrease the risk of SHF morbidity after β-blocker therapy; Disadvantage of beta-blocker indicated that the gene polymorphisms could not reduce the risk of SHF morbidity after β-blocker therapy.

On the other hand, in 260 patients with SHF, there were 66.5 percent of patients without β-blocker treatment, and 20.4 percent of patients received metoprolol treatment, 10.0 percent of patients had bisoprolol, but carvedilol user only occupied 3.1 percent of SHF patients. It appeared to evidently different of beta-blockers usage between non-SHF group and SHF group (see Table 3). Further, we assessed whether GRK5 and ADRB1 polymorphisms were associated with the risk of SHF morbidity among β-blocker user, our findings showed that the naive patients carrying genotype ADRB1 Gly389Gly did not reduce significantly the risk of SHF morbidity after β-blocker therapy (Odd ratio (OR), 0.48; 95% confidence interval, 0.08-3.00; P = 0.415) except for other ADRB1 and GRK5 genotypes (see Figure 2).

## Discussion

The findings of this study were that (1) GRK5 Leu41Leu genotype was not found in our Chinese population (see Figure 1); (2) Neither ADRB1 nor GRK5 variants presented independently associated with the risk of SHF morbidity; (3) most ADRB1 and GRK5 polymorphisms did reduce significantly the risk of SHF morbidity after β-blocker therapy except for those carrying genotype ADRB1 Gly389Gly.

The β1-adrenergic receptor is the predominant subtype expressed in the human heart and plays an important role in the physiologic and pathophysiologic regulation of the cardiovascular system [18]. Genetic polymorphisms of the major cardiac β-blocker target, the β1-adrenergic receptor, and a kinase that terminates its signaling, GRK5, can significantly impact HF outcomes, and that adjusting for these gene variants abrogates the apparent ethnic differences in β-blocker treatment effect on SHF development [19]. It was reported that the minor allele frequency of GRK5 Leu41 was 32.6% in blacks and 0% in whites [20]. Another study indicated that among 600 UK and Dutch participants, the prevalence of the ADRB1 genotypes was Arg389Arg 51.3%, Arg389Gly 40.2%, Gly389Gly 8.5% [21]. In our non-SHF Chinese population, the genotype frequencies of Gln41Gln, Gln41Leu and Leu41Leu were 0.985, 0.015 and 0 respectively, allele frequencies of Gln41 and Leu41 were 0.992 and 0.008, which is accordant with those American whites [20], suggesting that the GRK5 homozygous mutation probability were very rare in our population. On the other hand, the genotype frequencies of ADRB1 Arg389Arg, Arg389Gly and Gly389Gly were 0.508, 0.413 and 0.079 in our Chinese population, allele frequencies of the minor ADRB1 Gly389 were 0.285, which was similar to the reported allele frequencies in normal Caucasian American subjects (Caucasians, 0.12 to 0.25 and African American, 0.23 to 0.38 respectively) [19] and European whites [21].

In experimental mouse models, overexpression of β adrenergic receptors (β ARs) [22] or their Gas G protein signaling transducer [23] causes cardiac dilation and failure. Conversely, genetic ablation of the β ARs and Gas downstream effector, adenylylcyclase, preserves myocardial function after physiological stress [24]. GRK5-Leu41 accelerated isoproterenol-promoted β ARs desensitization, suggesting that, like β-blockers, it might protect hearts from the effects of persistent β AR stimulation, that is, cardiac dilation, ventricular hypertrophy and heart failure [25]. Unfortunately, we did not found the GRK5 homozygous mutation (GRK5 Leu41Leu) in our population, which prevented us from further exploring its protection of SHF.

Lately a meta-analysis report included that 2642 cases and 3136 controls in 12 case–control population studies provided data on the association between Arg389Gly polymorphisms and susceptibility to idiopathic dilated cardiomyopathy, finally found that no significantly elevated idiopathic dilated cardiomyopathy risk was associated with Arg389Gly polymorphisms for all genetic models in all populations [26]. Similarly White HL et al also reported that ADRB1 Arg389Gly polymorphisms were not relevant to heart failure events among the patients in MERIT-HF research center of the UK and Holland [21]. The present study found that the allele frequencies of whether GRK5 Gln41 and Leu41 or ADRB1 Arg389 and Gly389 were similarly distributed between non-SHF population and SHF population, suggesting that GRK5 and ADRB1 polymorphisms were not associated with SHF morbidity, further GRK5 and ADRB1 variants were not significantly independently associated with the risk of SHF morbidity (see Table 2). Thus our findings were consistent with the previous reports.

Given the reported associations between the β1 AR-Arg389 polymorphism and the response to β-blocker treatment with heart failure [9], the much higher norepinephrine affinity and adrenergic signal transduction capacity of Arg389 β1-AR may protect patients from excessive sympatholysis [27] that has been associated with increased mortality in β1 389-Gly carriers receiving bucindolol [28]. For other beta-blockers ADRB1 389Arg homozygotes may have greater improvements in LVEF compared with ADRB1 389Gly carriers [29,30], but no reduction in clinical endpoints has been demonstrated [12]. Latestly, bucindolol was associated with reductions in composite HF endpoints in those with the β1 389-Arg homozygous genotype [31]. Noticeably, the Arg389 variant of the β1-adrenergic receptor was associated with a greater response to metoprolol than that of Gly389 in young, male Chinese subjects [32]. In our study, most ADRB1 and GRK5 polymorphisms did decrease the risk of SHF morbidity after β-blocker therapy, but those carrying genotype ADRB1 Gly389Gly had not likelihood of benefit from β-blocker therapy (see Figure 2).

### Limitations

This was a cross-sectional data analysis, and it had better validate these findings prospectively. In addition, our data were derived from Han race in China, thus the findings were not generalized to other ethnicity in China.

## Conclusion

GRK5 Leu41Leu genotype was not found in our Chinese population, neither ADRB1 nor GRK5 variants presented independently associated with the risk of SHF morbidity, most ADRB1 and GRK5 polymorphisms did reduce the risk of SHF morbidity after β-blocker therapy, but those carrying genotype ADRB1 Gly389Gly had not likelihood of benefit from β-blocker therapy.
